# Rising prevalence of gestational diabetes mellitus and its associated risk factors in Makurdi, North-Central Region of Nigeria

**DOI:** 10.4314/ahs.v23i4.37

**Published:** 2023-12

**Authors:** Bruno Basil, Izuchukwu N Mba, Terna A Gav, Blessing K Myke-Mbata, Terrumun Z Swende, Simeon A Adebisi

**Affiliations:** 1 Department of Chemical Pathology, Benue State University, Makurdi, Nigeria; 2 Department of Obstetrics and Gynecology, Benue State University, Makurdi, Nigeria

**Keywords:** Pregnancy diabetes mellitus, prevalence, risk factor

## Abstract

**Background:**

The disease burden of gestational diabetes mellitus (GDM) in sub-Saharan African region have been on the rise. Proper assessment of current prevalence of GDM may inform policy changes and management approach for improved care delivery.

**Objective:**

To determine the current prevalence of Gestational Diabetes Mellitus (GDM) and evaluate its major risk factors amongst pregnant women in Makurdi, North-Central Nigeria.

**Method:**

This was a multi-center hospital-based prospective observational study. Maternal characteristics and clinical risk factors for GDM in a cohort of 281 pregnant women at 9 to 16 weeks gestational age was evaluated. The one-step 75g oral glucose tolerance test (OGTT) was carried out at 24 to 28 weeks of gestation.

**Result:**

Of the 356 women recruited, 281 (79.8%) completed the study. The GDM prevalence in the cohort was 16.7%. Increased early pregnancy BMI (adjusted OR = 1.154, 95% CI = 1.080 – 1.233, p<0.001) and presence of family history of diabetes mellitus (adjusted OR = 0.482, 95% CI = 0.233 – 0.997, P<0.05) were independent risk factors for GDM in the cohort.

**Conclusion:**

Increasing maternal age and early pregnancy BMI amongst other possible reasons, may account for the rising prevalence of GDM in the region.

## Introduction

Dysglycaemia in pregnancy which occurs as a result of Gestational Diabetes Mellitus (GDM) is associated with high risk of various complications both for the mother and foetus.^1^, ^2^ It alters the foetal milieu predisposing to possible epigenetic changes which may increase the risk of chronic metabolic diseases later in adult life.^3^ In addition to many other possible complications, this may also predispose the mother to increased risk of developing overt Type 2 diabetes mellitus (T2DM) later on.^2^ Gestational diabetes mellitus, is one of the commonest medical complications of pregnancy. It occurs when pancreatic beta-cells can no longer compensate for increasing insulin resistance 4 and is defined as any degree of glucose intolerance with onset, or first recognition during pregnancy.^5^ This definition does not exclude undiagnosed diabetes mellitus detected for the first time in the index pregnancy.^6^

The global prevalence of GDM is estimated to be 8.3%.^7^ In African, Hispanic, Indian, and Asian women prevalence are higher compared to Caucasian women.^8^ GDM prevalence in sub-Saharan Africa (SSA) is variable. Prevalence as low as 3.7% have been reported in some rural African settings^9^ while a crude prevalence of 13.9% was found amongst women at high risk for GDM in an urban population in Nigeria.^10^ These variable reports suggests the possibility of an increase in the prevalence of GDM which may have occurred over time.^11^–^15^ This may be as a result of increasing maternal age, body mass index (BMI), lifestyle changes corresponding to increasing incidence of type 2 diabetes mellitus (T2DM), changes in diagnostic criteria and/or changing definitions of GDM amongst other likely reasons.^16^

Common risk factors for GDM and associated complications include increasing maternal age, previous GDM, previous hyperglycaemia, increasing maternal BMI, family history of diabetes in first degree relative, previous large for gestational age pregnancy, polycystic ovarian syndrome (PCOS), previous perinatal loss, multiple pregnancies and use of medications such as antipsychotics and corticosteroids.^6^, ^17^ However, the short- and long-term maternal and foetal complications associated with GDM^1^–^3^ may be significantly reduced via proper identification and management, especially if instituted early in pregnancy.^18^

It is suggested that there has been remarkable increase in the prevalence and disease burden of GDM over time in various sub-Saharan African populations, 19–22 but no documented evidence of such changes in the North-Central region of Nigeria exists based on information available to the investigators. A proper assessment of the prevalence of GDM would inform policy decisions aimed towards improved identification and management of GDM,^23^ as well as reduction of its disease burden in the study area. This study was aimed at assessing and documenting the current prevalence of GDM against previously reported rates, as well as evaluating the major risk factors predisposing to the disease in the study population.

## Methodology

### Study design and setting

This was a multiple hospital-based cross-sectional and observational study which was conducted at the antenatal clinics of Benue State University Teaching Hospital (BSUTH), Family Support Programme Clinic, Federal Medical Centre (FMC), First Fertility Hospital and Pishon Women Hospital all in Makurdi, Nigeria between June 2018 and February 2019 (an 8-month period).

Inclusion Criteria were; pregnant women between 9 – 16 weeks gestational age as determined by early pregnancy abdominal ultra-sound scan, no known history of diabetes mellitus prior to index pregnancy, as well as Informed and consenting participants.

Exclusion Criteria were; known diabetics prior to index pregnancy, acutely or chronically ill patients (associated with stress-induced derangement in glucose control which will interfere with OGTT), medications known to affect glucose metabolism like glucocorticoids, those booked outside 9 – 16 weeks gestational age and non-consenting subjects.

A total 356 women who met the inclusion criteria were recruited into the study at their 9 to 16 weeks of gestation. However, only 281 who completed the study (OGTT at 24 to 28 weeks gestation) were included in the statistical analysis. Participants who met the inclusion criteria were selected using a computer-generated random number table from pregnant women attending antenatal clinic at the study sites after informed written consent had been obtained. Pregnant women known to be acutely or chronically ill, diabetic, on steroids or booked outside 9 – 16 weeks gestational age were excluded from the study. Also, participants with missing data were excluded from the study during statistical analysis.

### Data collection

Data on demographic characteristics and relevant maternal clinical risk factors for GDM of consenting participants was obtained from antenatal booking records and by use of a validated and structured study proforma.

### Diagnosis of GDM

At 24 – 28 weeks gestational age participants were subjected to OGTT with an oral load of 75 gram of anhydrous glucose after ensuring they maintain normal diet and activity for at least 3 days prior to testing. Samples for blood glucose assay were collected into vacutainer tubes with fluoride oxalate, separated within 20 minutes of collection and each batch was analysed immediately after collection.

The diagnosis of GDM was according to the updated international diagnostic criteria based on the International Association of Diabetes in Pregnancy Study Group (IADPSG) diagnostic guideline for universal testing of women [24] between 24 – 28 weeks gestation. GDM was diagnosed if fasting blood glucose level ≥ 5.1mmol/L, and/or 1-hour post-75g blood glucose level ≥ 10.0mmol/L, and/or 2-hour post-75g blood glucose level ≥ 8.5mmol/L.

### Statistical analyses

Data analysis was done using the Statistical Package for Social Sciences (SPSS) version 21. Categorical variables like history of first-degree relatives with DM, previous foetal macrosomia, etc., were represented in binary form and reported as percentages while continuous variables like age and BMI were expressed as mean ± SD. Comparison of means of continuous independent variables in both GDM and non-GDM participants was done using the student's T-test while the Chi square test was used for categorical independent variables. p-values < 0.05 were considered statistically significant in both univariate and multivariate analyses. Factors found to be statistically significant in univariate analysis were included in a multivariate model to identify independent risk factors for GDM after adjusting for potential confounders.

### Ethical consideration

Ethical approval was obtained from the Health Research and Ethics Committees of BSUTH Makurdi before commencement of the study. The standard requirements of the Ethics Committee of the BSUTH where this work was done was satisfactorily met. BSUTH is the apex health facility in Makurdi, its ethical clearance was accepted by the management of the other study sites. The aim and importance of the study, procedures involved, as well as the risks and benefits associated with participation in the study was explained to each participant prior to recruitment. They were allowed to raise concerns where required and clarifications were proffered. Participation in the study was entirely voluntary and they were made to understand that they may opt out of the study at any point without affecting the level of care they would receive from the hospital. Informed consent so obtained was individualized and documented as signified by the participant's signature and that of a witness.

Participants were allotted number codes to ensure confidentiality after providing an informed and written consent for inclusion in the study. Data from the study proforma and results obtained from analysis of serum sample collected from the participants were kept confidential by filing in locked spaces.

## Result

During the 8 months period of the study, a total of 356 women who met the inclusion criteria were recruited, but only 281 (79.8%) who were subsequently tested for GDM at 24 to 28 weeks of gestation were included in the study. Forty-seven (16.7%) of the women had GDM based on result of one-step 75g OGTT diagnostic method while 234 (83.3%) were without GDM.

Table 1 shows the socio-demographic and clinical characteristics of the participants. There were no statistically significant differences between GDM and non-GDM participants with regards to age groups, ethnicity, educational status, religion, parity and blood pressures.

**Table 1:** Some socio-demographic and clinical characteristics of the study participants

Characteristics	Total (n=281) Mean±SD or n (%)	GDM (n=47) Mean±SD or n (%)	Non-GDM (n=234) Mean±SD or n (%)	p-value
Age groups (years)				
< 25	52 (18.5)	4 (7.7)	48 (92.3)	0.088
25 – 35	202 (71.9)	36 (17.8)	166 (82.2)	
> 35	27 (9.6)	7 (25.9)	20 (74.1)	
Ethnic groups				
Tiv	156 (55.5)	33 (21.2)	123 (78.8)	0.066
Idoma	62 (22.0)	4 (6.5)	58 (93.5)	
Igbo	33 (11.7)	6 (18.2)	27 (81.8)	
Others	30 (10.7)	4 (13.3)	26 (86.7)	
Educational status				
Uneducated	66 (23.5)	14 (21.2)	52 (78.8)	0.250
Primary	58 (20.6)	13 (22.4)	45 (77.6)	
Secondary	65 (23.1)	8 (12.3)	57 (87.7)	
Tertiary	92 (32.7)	12 (13.0)	80 (87.0)	
Religion				
Christian	237 (84.3)	40 (16.9)	197 (83.1)	0.538
Muslim	44 (15.7)	7 (15.9)	37 (84.1)	
Parity				
Primigravida	94 (33.5)	11 (11.7)	83 (88.3)	0.074
Multigravida	187 (66.5)	36 (19.3)	151 (80.7)	
Blood Pressure				
Systolic (mmHg)	108.1 ± 10.9	107.9 ± 10.6	108.2 ± 10.9	0.690
Diastolic (mmHg)	72.6 ± 10.8	72.5 ± 10.8	73.0 ± 10.4	0.961

Table 2 shows the relationship between maternal clinical risk factors for GDM and development of the disease. The mean age of the participants was 29.2 ± 4.8 years. Women who developed GDM had a significantly higher mean age of 31.2 ± 4.6 years compared to those without GDM (28.7 ± 4.8 years) (p = 0.001). The mean early pregnancy BMI of the study participants was 28.4 ± 5.3 kg/m^2^. Those who developed GDM had a statistically significant higher BMI (32.1 ± 4.9 kg/m^2^) compared to those who did not (27.6 ± 5.1 kg/m^2^) (p < 0.001). Statistically significant differences between GDM versus non-GDM women were also noted in some maternal clinical risk factors for GDM like having a previous history of GDM (83.3% vs 16.7%; p=0.001), first degree relatives with diabetes mellitus (68.9% vs 31.1%; p=0.001) and history of foetal macrosomia (71.0% vs 29.0%; p=0.004) on univariate analysis, while participants with history of perinatal loss, multiple pregnancies and/or pre-eclampsia in their previous pregnancies showed no statistically significant differences between GDM and non-GDM participants. However, after a multivariate logistic regression analysis with sequential backward elimination and adjusting for confounding variables, only early pregnancy BMI (adjusted OR = 1.154, 95% CI = 1.080 – 1.233, p<0.001) and family history of diabetes mellitus (adjusted OR = 0.482, 95% CI = 0.233 – 0.997, p=0.049) were independent risk factors for GDM in the cohort.

**Table 2:** Risk factors for gestational diabetes mellitus in the cohort

Characteristics	Total (n=281) Mean±SD or n (%)	GDM Mean±SD or n (%)	Non-GDM Mean±SD or n (%)	p-value
Age (years)	29.2 ± 4.8	31.2 ± 4.6	28.7 ± 4.8	0.001*
Early pregnancy BMI (kg/m^2^)	28.4 ± 5.3	32.1 ± 4.9	27.6 ± 5.1	<0.001*
Previous history of GDM	6 (2.1)	5 (83.3)	1 (16.7)	0.001*
History of first-degree relation with diabetes mellitus	61 (21.7)	42 (68.9)	19 (31.1)	0.001*
History of perinatal loss	37 (13.2)	28 (75.7)	9 (24.3)	0.138
History of multiple pregnancies	19 (6.8)	13 (68.4)	6 (31.6)	0.076
Foetal macrosomia in previous pregnancies	62 (22.1)	44 (71.0)	18 (29.0)	0.004*
History of Pre-eclampsia	15 (5.3)	11 (73.3)	4 (26.7)	0.229

## Discussion

The pooled prevalence of GDM based on updated international diagnostic criteria in Africa is 13.6% (95% CI: 11.0 – 16.2) while in the sub-Saharan African region, an estimated prevalence of 14.3% has been reported.^20^ In Nigeria, a systematic review reported a pooled prevalence of 11.0% (95% CI: 8.0 – 13.0),^25^ and a prevalence of 8.3% has been earlier reported in a similar population of pregnant women in the North-Central region of Nigeria.^26^ The high prevalence of GDM in this study (16.7%) based on updated criteria may be in keeping with the high burden of the disease in the region and its progressive increase over time.^11^, ^19^ This is consistent with a recent systematic review and meta-analysis that reports a GDM prevalence of 16.0% (95% CI: 8.0 – 25.0) in the sub-Saharan African region.^25^ This high prevalence from both studies may be attributable to increasing maternal age and BMI, changes in diagnostic criteria and/or definition of GDM, as well as other lifestyle changes linked to urbanization which has also been associated with increasing prevalence of T2DM in the sub-Saharan African region.^27^

An important determinant of GDM prevalence is the chosen diagnostic approach. Reports from previous studies showed that the IADPSG criteria has better sensitivity than other diagnostic criteria and is capable of detecting more women with GDM.^28^, ^29^ The prevalence of GDM in the index study was far higher that of a previous hospital-based study carried out in the same geographical region where a point prevalence of 8.3% was obtained.[26] Despite the mean age of the participants in that study being similar to that in the index study, remarkable difference in prevalence is mostly accounted for by the chosen diagnostic methods.^30^, ^31^ In that study, a 75g OGTT was done after an initial screening via 1-h 50g glucose challenge test (GCT). This two-step approach has lower sensitivity compared to the one-step 75g OGTT approach used in this study 32, 33

However, use of same diagnostic approach yielded a lower prevalence rate (11.6%) in a previous study of a cohort of pregnant women receiving ante-natal care in Lagos, South-West Nigeria and higher in a more recent study in South-East Nigeria (38.0%) when compared to the index study.^34^, ^35^ This may be due to progressive increase in GDM prevalence as a result of changes in maternal clinical and demographic characteristics over time, higher detection rates as a result of improved diagnosis of GDM, and/or sociocultural, environmental, and economic factors, resulting in differences in accessing ANC services, as well as other population lifestyle differences.

Another factor that may impact GDM prevalence is changing definitions of GDM. The IADPSG based diagnostic criteria utilized in this study usually reports a higher prevalence because it includes hyperglycaemia levels categorized as Diabetes in Pregnancy (DIP) by the 2013 WHO criteria in the definition of GDM, provided it was first recognized in pregnancy. For instance, a previous study aimed at determining the prevalence of GDM in South-East Nigeria reported a higher prevalence (38.0%) using the IADPSG criteria compared to the 2013 WHO criteria (35.9%). The difference is primarily because a further 2.1% was classified as having DIP by the 2013 WHO criteria,^35^ while all subjects without prior history of diabetes mellitus identified to have any degree of hyperglycemia in pregnancy were classified as GDM by the IADPSG criteria.

Other factors like ethnicity, increasing maternal age and BMI may have contributed to the increase in GDM prevalence. Ethnicity (black race) have been found to be associated with higher levels of insulin resistance 36–38 and contributes to the high incidence of GDM amongst Africans. However, key factors that may affect GDM prevalence in the region are the increasing incidence of obesity^39^ and the progressive increase in maternal age.^13^, ^40^ For instance, the mean age of the participants in this study is 29.2 ± 4.8 years which is higher than the recommended cut-off of 25 years indicative of a high risk of GDM from previous studies.^41^, ^42^ Similarly, the mean early pregnancy BMI of the participants (28.4 ± 5.3 kg/m^2^) fell within the overweight category (25.0 – 29.9 kg/m^2^) and BMI greater than 25 kg/m2 has been strongly associated with high risk of GDM.^43^ This has been reported in a recent Chinese study where early pregnancy BMI in the overweight/obese category was associated with increased risk of maternal GDM.^44^ This higher BMI and obesity has been known to strongly correlate with increased insulin resistance and glucose intolerance^45^ which in pregnancy causes GDM.

Furthermore, this study also assessed the major risk factors predisposing to GDM in the study population. Risk factors associated with GDM development in this study are as reported in previous studies carried out in sub-Saharan Africa. 11, 19, 20, 26, 46 However, only increased early pregnancy BMI and having a family history of diabetes mellitus were significant independent risk factors for GDM in the cohort with an adjusted OR of 1.154 (95% CI = 1.080 – 1.233, p < 0.001) and 0.482 (95% CI = 0.233 – 0.997, p<0'05 respectively. When compared with a previous study from a similar population within the region, previous foetal macrosomia was the only independent predictor of GDM in that study (adjusted OR 11.1; 95 % CI 2.93 – 42.12, p<0.001. Difference in these findings as well as other risk factors in the multivariate model being insignificant is possibly due to the shorter duration of the study, smaller sample sizes and/or presence of some unknown confounding factors in both cohorts. A larger study would be required to identify other risk factors for GDM in the region.

This study carried out a one-time point assessment for GDM (24 – 28 weeks of gestation) and participants were not followed up to assess for birth outcome and subsequent post-delivery testing for GDM. As such, the study cannot reliably claim a causal relationship between identified risk factors and GDM development. We recommend further studies with a larger sample size and study duration to better assess risk factors for GDM development throughout the period of pregnancy and the post-delivery period in the region.

## Conclusion

There has been a remarkable rise in the prevalence of GDM in the North-Central region of Nigeria over time which may be attributable to increasing maternal age and BMI, changes in diagnostic criteria and/or definition of GDM, and lifestyle changes amongst other possible reasons.

## References

[1] Boyd EM1, Thomas AB, Donald RC, Alberto de L, David BD, David RH (2007). Summary and Recommendations of the Fifth International Workshop-Conference on Gestational Diabetes Mellitus. Diabetes Care.

[2] Bellamy L, Casas JP, Hingorani AD, Williams D (2009). Type 2 diabetes mellitus after gestational diabetes: a systematic review and meta-analysis. Lancet.

[3] Hanson MA, Gluckman PD (2014). Early Developmental Conditioning of Later Health and Disease: Physiology or Pathophysiology?. Physiol Rev.

[4] Catalano PM (1994). Carbohydrate metabolism and gestational diabetes. Clin Obstet Gynecol.

[5] Alfadhli EM (2015). Gestational diabetes mellitus. Saudi Med J.

[6] Nankervis A, McIntyre HD, Moses R, Ross GP, Call-away L, Porter C (2008). ADIPS Consensus Guidelines for the Testing and Diagnosis of Gestational Diabetes Mellitus in Australia. Australas Diabetes Pregnancy Soc.

[7] International Diabetes Federation (2014). IDF Diabetes Atlas.

[8] American Diabetes Association (2011). Diagnosis and Classifixation of Diabetes Mellitus. Diabetes Care.

[9] Seyoum B, Kiros K, Haileselase T, Leole A (1999). Prevalence of gestational diabetes mellitus in rural pregnant mothers in northern Ethiopia. Diabetes Research and Clin Pract.

[10] Kuti MA, Abbiyesuku FM, Akinlade KS, Akinosun OM, Adedapo KS, Adeleye JO (2011). Oral glucose tolerance testing outcomes among women at high risk for gestational diabetes mellitus. J Clin Pathol.

[11] Mwanri AW, Kinabo J, Ramaiya K, Feskens EJM (2015). Gestational diabetes mellitus in sub-Saharan Africa: systematic review and meta regression on prevalence and risk factors. Trop Med Int Health.

[12] Lavery JA, Friedman AM, Keyes KM, Wright JD, Ananth C V (2017). Gestational diabetes in the United States: temporal changes in prevalence rates between 1979 and 2010. BJOG An Int J Obstet Gynaecol.

[13] Ferrara A (2007). Increasing prevalence of gestational diabetes mellitus: A public health perspective. Diabetes Care.

[14] Dabelea D, Snell-Bergeon JK, Hartsfield CL, Bischoff KJ, Hamman RF, McDuffie RS (2005). Increasing Prevalence of Gestational Diabetes Mellitus (GDM) Over Time and by Birth Cohort. Diabetes Care.

[15] Dornhorst A, Paterson CM, Nicholls JSD, Wadsworth J, Chiu D C, Elkeles R S (1992). High Prevalence of Gestational Diabetes in Women from Ethnic Minority Groups. Diabet Med.

[16] Queensland Clinical Guidelines (2015). Gestational Diabetes Mellitus.

[17] Doherty E, Kingsland M, Wiggers J, Wolfenden L, Hall A, McCrabb S (2022). The effectiveness of implementation strategies in improving preconception and antenatal preventive care: a systematic review. Implement Sci Commun.

[18] Seshiah V, Cynthia A, Balaji V, Balaji Madhuri S, Ashalata S, Sheela Rajan (2008). Detection and care of women with gestational diabetes mellitus from early weeks of pregnancy results in birth weight of newborn babies appropriate for gestational age. Diabetes Res Clin Pract.

[19] Natamba BK, Namara AA, Nyirenda MJ (2019). Burden, risk factors and maternal and offspring outcomes of gestational diabetes mellitus (GDM) in sub-Saharan Africa (SSA): A systematic review and meta-analysis. BMC pregnancy and childbirth.

[20] Muche AA, Olayemi OO, Gete YK (2019). Prevalence and determinants of gestational diabetes mellitus in Africa based on the updated international diagnostic criteria: A systematic review and meta-analysis. Arch Public Heal.

[21] Veeraswamy S, Vijayam B, Gupta VK, Kapur A (2012). Gestational diabetes: The public health relevance and approach. Diabetes Research and Clinical Practice.

[22] Adebisi SA, Oparinde DP, Olarinoye JK, Aboyeji PA, Ogunro PS (2004). Gestational diabetes mellitus: diagnostic and management approach. Postgraduate Doctor Caribbean.

[23] Adebisi SA, Oghagbon K, Jimoh AK, Akande T, Olarinoye JK (2009). Quality of diabetic care in a tertiary health care facility in Ilorin, Nigeria. Diabetol Croat.

[24] Gupta Y, Kalra B, Baruah MP, Singla R, Kalra S (2015). Updated guidelines on screening for gestational diabetes. International Journal of Women's Health.

[25] Azeez TA, Abo-Briggs T, Adeyanju AS (2021). A systematic review and meta-analysis of the prevalence and determinants of gestational diabetes mellitus in Nigeria. Indian Journal of Endocrinology and Metabolism.

[26] Anzaku AS, Musa J (2013). Prevalence and associated risk factors for gestational diabetes in Jos, North-central, Nigeria. Arch Gynecol Obstet.

[27] Ojuka EO, Goyaram V (2014). Increasing prevalence of type 2 diabetes in sub-Saharan Africa: not only a case of inadequate physical activity. Medicine and sport science.

[28] Shang M, Lin L (2014). IADPSG criteria for diagnosing gestational diabetes mellitus and predicting adverse pregnancy outcomes. J Perinatol.

[29] Brown FM, Wyckoff J (2017). Application of One-Step IADPSG Versus Two-Step Diagnostic Criteria for Gestational Diabetes in the Real World: Impact on Health Services, Clinical Care, and Outcomes. Current Diabetes Reports.

[30] Pastakia SD, Njuguna B, Onyango BA, Washington S, Christoffersen-Deb A, Kosgei WK (2017). Prevalence of gestational diabetes mellitus based on various screening strategies in western Kenya: A prospective comparison of point of care diagnostic methods. BMC Pregnancy Childbirth.

[31] Dias T, Siraj SHM, Aris IM, Li LJ, Tan KH (2018). Comparing different diagnostic guidelines for gestational diabetes mellitus in relation to birthweight in Sri Lankan women. Front Endocrinol (Lausanne).

[32] American Diabetes Association (2004). Position statement - Gestational diabetes mellitus. Diabetes Care.

[33] Berghella V, Caissutti C, Saccone G, Khalifeh A (2019). The One Step approach for diagnosing gestational diabetes is associated with better perinatal outcomes than the Two Step approach: evidence of randomized clinical trials. Am J Obstet Gynecol.

[34] Olarinoye JK, Ohwovoriole AE, Ajayi GO (2004). Diagnosis of gestational diabetes mellitus in Nigerian pregnant women - Comparison between 75G and 100G oral glucose tolerance tests. West Afr J Med.

[35] Onyenekwe BM, Young EE, Nwatu CB, Okafor CI, Ugwueze CV, Chukwu SN (2019). Prevalence of Gestational Diabetes in South East Nigeria Using the Updated Diagnostic Guidelines. Int J Diabetes Metab.

[36] Fuller-Rowell TE, Homandberg LK, Curtis DS, Tsenkova VK, Williams DR, Ryff CD (2019). Disparities in insulin resistance between black and white adults in the United States: The role of lifespan stress exposure. Psychoneuroendocrinology.

[37] Kodama K, Tojjar D, Yamada S, Toda K, Patel CJ, Butte AJ (2013). Ethnic differences in the relationship between insulin sensitivity and insulin response: A systematic review and meta-analysis. Diabetes Care.

[38] Mocarski M, Savitz DA (2012). Ethnic differences in the association between gestational diabetes and pregnancy outcome. Matern Child Health J.

[39] Steyn NP, Mchiza ZJ (2014). Obesity and the nutrition transition in Sub-Saharan Africa. Ann N Y Acad Sci.

[40] Muganyizi PS, Kidanto HL (2009). Impact of change in maternal age composition on the incidence of Caesarean section and low birth weight: Analysis of delivery records at a tertiary hospital in Tanzania, 1999-2005. BMC Pregnancy Childbirth.

[41] Lao TT, Ho LF, Chan BCP, Leung WC (2006). Maternal age and prevalence of gestational diabetes mellitus. Diabetes Care.

[42] Danilenko-Dixon DR, Van Winter JT, Nelson RL, Ogburn PL (1999). Universal versus selective gestational diabetes screening: Application of 1997 American Diabetes Association recommendations. American Journal of Obstetrics and Gynecology.

[43] Garrison A (2015). Screening, diagnosis, and management of gestational diabetes mellitus. Am Fam Physician.

[44] Chen YT, Zhang T, Chen C, Xia YY, Han TL, Chen XY (2021). Associations of early pregnancy BMI with adverse pregnancy outcomes and infant neurocognitive development. Sci Rep.

[45] Wondmkun YT (2020). Obesity, insulin resistance, and type 2 diabetes: Associations and therapeutic implications. Diabetes, Metabolic Syndrome and Obesity: Targets and Therapy.

[46] Egbe TO, Tsaku ES, Tchounzou R, Ngowe MN (2018). Prevalence and risk factors of gestational diabetes mellitus in a population of pregnant women attending three health facilities in Limbe, Cameroon: A cross-sectional study. Pan Afr Med J.

